# Hypotonic Shock Modulates Na^+^ Current via a Cl^-^ and Ca^2+^/Calmodulin Dependent Mechanism in Alveolar Epithelial Cells

**DOI:** 10.1371/journal.pone.0074565

**Published:** 2013-09-03

**Authors:** André Dagenais, Marie-Claude Tessier, Sabina Tatur, Emmanuelle Brochiero, Ryszard Grygorczyk, Yves Berthiaume

**Affiliations:** 1 Institut de Recherches Cliniques de Montréal (IRCM), Montreal, Quebec, Canada; 2 Département de Médecine, Université de Montréal, Montreal, Quebec, Canada; 3 Centre de Recherche du CHUM (CRCHUM), Centre Hospitalier de l’Université de Montréal, Montreal, Quebec, Canada; University of Geneva, Switzerland

## Abstract

Alveolar epithelial cells are involved in Na^+^ absorption via the epithelial Na^+^ channel (ENaC), an important process for maintaining an appropriate volume of liquid lining the respiratory epithelium and for lung oedema clearance. Here, we investigated how a 20% hypotonic shock modulates the ionic current in these cells. Polarized alveolar epithelial cells isolated from rat lungs were cultured on permeant filters and their electrophysiological properties recorded. A 20% bilateral hypotonic shock induced an immediate, but transient 52% rise in total transepithelial current and a 67% increase in the amiloride-sensitive current mediated by ENaC. Amiloride pre-treatment decreased the current rise after hypotonic shock, showing that ENaC current is involved in this response. Since Cl^-^ transport is modulated by hypotonic shock, its contribution to the basal and hypotonic-induced transepithelial current was also assessed. Apical NPPB, a broad Cl^-^ channel inhibitor and basolateral DIOA a potassium chloride co-transporter (KCC) inhibitor reduced the total and ENaC currents, showing that transcellular Cl^-^ transport plays a major role in that process. During hypotonic shock, a basolateral Cl^-^ influx, partly inhibited by NPPB is essential for the hypotonic-induced current rise. Hypotonic shock promoted apical ATP secretion and increased intracellular Ca^2+^. While apyrase, an ATP scavenger, did not inhibit the hypotonic shock current response, W7 a calmodulin antagonist completely prevented the hypotonic current rise. These results indicate that a basolateral Cl^-^ influx as well as Ca^2+^/calmodulin, but not ATP, are involved in the acute transepithelial current rise elicited by hypotonic shock.

## Introduction

In the lungs, the transepithelial Na^+^ transport plays an important role in modulating the amount of fluid lining the respiratory epithelium [1,2]. This process is crucial at birth for fluid removal from the airspaces [3] and in the adulthood, for lung liquid clearance during acute lung injury (ALI) [1,4]. The Epithelial Na^+^ channel (ENaC) composed of 3 subunits (α, β, γ) [5,6] expressed in type I and type II alveolar epithelial cells, is the main channel involved in this process [1,2]. This has been demonstrated experimentally in αENaC KO mice where pups, unable to reabsorb lung fluid, die shortly after birth [7]. In adult mice expressing a lower amount of αENaC in the lung, the wet to dry ratio was increased in 2 models of lung injury [8], in comparison to wild type controls.

Several factors have been shown to modulate ENaC expression and activity in the lungs, including purinergic signaling [9–11], glucocorticoid [12–16], protease activation [10,17,18] and membrane recycling or lysosomal degradation of the channel [19]. Since transepithelial Na^+^ transport involves activity of the Na/K-ATPase expression at the basolateral side, factors that regulate the sodium pump or its membrane insertion have an impact on the Na^+^ transport system [14,20]. The ENaC mediated transepithelial Na^+^ transport is also influenced by K^+^ and Cl^-^ channels since modulating the membrane potential changes the driving force for Na^+^ [21–23].

Reduced extracellular osmolarity induces a hypotonic shock which promotes water influx and cell swelling [24]. In renal A6 epithelial cells, hypotonic shock has been shown to gradually increase the ENaC-mediated transepithelial current via tyrosine kinase [25], Cl^-^ [26] and Ca^2+^/calmodulin [27,28] dependent mechanisms. In contrast to kidney epithelial cells that are challenged constantly with tonicity changes in the nephron, alveolar epithelial cells are not exposed to hypotonic shock except in fresh water drowning. Nevertheless, all cells have the ability to modulate their cell volume in response to extracellular or intracellular osmolyte variation [24]. For alveolar epithelial cells, the transepithelial Na^+^ transport *per se* leads to cell volume increase because of transepithelial and transcellular Na^+^ and H_2_O flux. Hypo-osmotic challenge is an interesting experimental tool to study the nature of the channels and ionic transporters expressed in a cell type and to study how change in cell volume and mechanical stress affect epithelial physiology. We reported previously that hypotonic shock promotes ATP secretion in A549 alveolar epithelial cells, and elevates cytoplasmic Ca^2+^ [29–31], two factors that could modulate transepithelial ENaC current. In the present work, we investigated if hypotonic shock could modulate ENaC-mediated transepithelial current in rat alveolar epithelial cells and if Ca^2+^, purinergic signaling or Cl^-^ could play a role in this process. We found that hypotonic shock acutely increased total and Na^+^ short circuit current (*I*_*sc*_). Testing physiological buffers with different ionic compositions and inhibitors for different channels and transporters, we established that Cl^-^ and the potassium/chloride co-transporter (KCC) play an important role in modulating the basal and hypotonic-induced transepithelial current. Although ATP was secreted during hypotonic shock, purinergic signaling was not a factor in the current rise after hypotonic shock.

## Materials and Methods

### Ethic statements

AEC were isolated from rat lungs according to a procedure approved by our institutional animal care committee (IACC) of Institut de recherches cliniques de Montréal (IRCM) in accordance with the Canadian Council of Animal Care (CCAC) standards.

### Material

Amiloride, ATP (Adenosine 5′-triphosphate disodium salt), bumetanide (3-(Aminosulfonyl)-5-(butylamino)-4-phenoxybenzoic acid), clofilium tolysate (4-Chloro-N,N-diethyl-N-heptylbenzenebutanaminium tosylate), DIOA (R-(+)-[(2-n-Butyl-6,7-dichloro-2-cyclopentyl-2,3-dihydro-1-oxo-1H-inden-5-yl) oxy] acetic acid), NPPB (5-nitro-2-(3-phenylpropylamino) benzoic acid), BAPTA-AM (1,2-Bis(2-aminophenoxy)ethane-N,N,N′,N′-tetraacetic acid tetrakis(acetoxymethyl ester)) and W7 (N-(6-aminohexyl)-5-chloro-1-naphthalenesulfonamide hydrochloride) were purchased from Sigma-Aldrich (Sigma-Aldrich, Oakville, ON, Canada).

### Cell isolation

Alveolar epithelial cells were isolated from male Sprague-Dawley rats (*Rattus norvegicus*) as described previously [32]. 175-200g male rats were anaesthetized by intraperitoneal injection of Pentobarbital (80-100 mg/kg). After thorachotomy and insertion of a blunt 18S needle into trachea, the animals were exsanguinated by incision of abdominal aorta. The lungs were perfused with perfusion solution (135 mM NaCl, 4.86 mM glucose, 5.3 mM KCl, 1.9mM CaCl_2_, 1.3 mM MgSO_4_, 10 mM HEPES, 2.65 mM phosphate buffer pH 7.4) via pulmonary artery until they appear white. After excision and removal from thoracic cavity, the lungs were digested at 37^o^C with elastase injected into trachea. Alveolar epithelial cells were purified by a differential adherence technique on bacteriological plastic plates coated with rat I_g_G [32]. On day 0, the cells were plated at 1X10^6^ cells/cm^2^ on polycarbonate membrane inserts (Corning Transwell, Thermo, Fisher Scientific, Nepean, ON, Canada). They were then cultured at 37^o^C with 5% CO_2_ in a humidified incubator in MEM (Life Technologies, Burlington, ON, Canada) supplemented with 10% FBS (GIBCO BRL, Life Technologies), 0.08 mg/l gentamycin, 0.2% NaHCO_3_, 0.01 M HEPES, 2 mM L-glutamine and Septra (3 µg/ml trimethoprim with 17 µg/ml sulfamethoxazole). The medium was changed after 48h in culture, but without septra. The electrophysiological measurements as well as ATP and Ca^2+^ assays were performed at day 4 of culture.

### Electrophysiology

*I*_*sc*_ were measured at 37°C in cell monolayers grown 4 days on polycarbonate membrane inserts (24 mm diameter, catalogue #3412, Corning Transwell) placed horizontally in a modified Ussing chamber [10]. The physiological buffer for *I*_*sc*_ measurement contained 140 mM NaCl, 5 mM KCl, 1 mM MgCl_2_, 1 mM CaCl_2_, 10 mM glucose, 10 mM TES, pH 7.4 at 315 mOsm/kg. The Cl^-^ reduced buffer (Cl^-^(-)) was obtained by substituting Na gluconate instead of NaCl to decrease Cl^-^ from 159 mM to 9 mM. KCl was omitted to generate a buffer devoid of K^+^ (K^+^(-)). Voltage (calomel) and current (Ag/AgCl) electrode pairs were in contact with the apical and basolateral bathing solutions and connected via 2 M KCl/5% agarose bridges. *I*_sc_ were generated by a VCC MC2 voltage clamp amplifier (Physiological Instruments, San Diego, CA) linked to a 4sp PowerLab data acquisition and analysis system (ADInstruments, Grand Junction, CO). The currents were recorded and analysed with the PowerLabs Chart 5 software. Transepithelial resistance (R_te_) was calculated with the Ohm’s law by recording the current jump obtained after applying a 1-mV square pulse for 1 s every 10 s. To test the *I*_sc_ generated by 20% hypotonic shock, an appropriate volume of water was added to the apical and basolateral compartments. At the peak response (2-3 minutes), 1 µM amiloride was added to determine the ENaC-mediated currents while 100 µM amiloride was added to ascertain amiloride-sensitive *I*_*sc*_ mediated by the non-selective cationic channel (NSC) reported to be expressed in these cells [32–34]. To test how apical ATP modulates total and ENaC *I*_sc_, alveolar epithelial cells monolayers were mounted in a Ussing chamber, and when baseline *I*_*sc*_ was stable for at least 10 min, amiloride-sensitive *I*_*sc*_ was assessed by adding 1 µM amiloride on the apical monolayer side for 5 minutes. After washing out amiloride and returning to basal *I*_*sc*_, the cells were exposed to 100 µM ATP (apical and/or basolateral). Total and ENaC *I*_*sc*_ were recorded again after 5-min incubation with ATP.

### ATP efflux assay

After 4 days of culture on 12 mm permeant filters (Corning Transwell), alveolar epithelial cells were bathed in a physiological solution (140 mM NaCl, 5 mM KCl, 1 mM MgCl_2_, 1 mM CaCl_2_, 10 mM glucose, 10 mM TES, pH 7.4), free of FBS, to avoid interference with the luciferin/luciferase reaction. Because medium change could induce mechanical stress and ATP release, the cells were incubated for 4 h at 37^o^C to allow ATP to return to baseline. The cells were then agitated gently on a rocking platform at 37^o^C. This mixed the medium covering the cells and minimized the effect of the unstirred layer. Basal ATP was quantified at time 0 (T_0_) and after the 4-h pre-incubation period. Then, water was added gently to generate 20% hypotonic shock compared to the control condition. An equivalent volume of isotonic buffer was provided for control isotonic samples. Aliquots of the medium were collected at 1, 5, 15 and 60 min after application of isotonic or hypotonic media. Extracellular ATP concentration was measured by luciferase bioluminescence in a TD-20/20 luminometer (Turner Designs, Sunnyvale, CA). For this assay, 50 µl of the luciferin/luciferase reagent (1 mg/ml) (catalogue # F-3641, Sigma-Aldrich, Oakville, Ont, Canada) were mixed with a 50 µl aliquot of extracellular medium immediately before reading. Luminescence signals were integrated for 10 s (Turner TD-20/20 luminometer) and expressed in relative light units. ATP concentration of the samples was estimated by regression of the light signal compared to a standard curve with known amounts of ATP. Concentrations were expressed in moles/1X10^6^ cells.

### Fura 2 [Ca^2+^]_i_ imaging

Ca^2+^ was monitored in cells after 20% hypotonic shock as reported previously [29,30]. Briefly, cells were loaded (1 h, room temperature) with 10 µM Fura-2-AM in physiological buffer (140 mM NaCl, 5 mM KCl, 1 mM MgCl_2_, 1 mM CaCl_2_, 10 mM glucose, 10 mM TES, pH 7.4) containing 0.02% Pluronic F127 and 2.5 mM of probenecid, followed by 30-min de-esterification in physiological solution containing probenecid. For calcium imaging, coverslips with Fura-2-loaded cells were mounted in the imaging/perfusion chamber attached to the heated platform (Warner Instruments, Hamden, CT) on the stage of an inverted microscope (Nikon TE300, Missisauga, ON). The cells were exposed to alternate (200 ms) illumination at 340 nm and 380 nm with a high-pressure mercury lamp (100 W) via interference filters (Chroma Technology, Brattleboro, VT) mounted on a filter wheel (Sutter Lambda 10-C, Sutter Instrument, Novato, CA) with a dichroic mirror (510/540 nm, Chroma Technology). Fluorescent images were recorded at 15- to 60-s intervals by digital camera. Fura-2 measurements are presented as the *F*_*340*_ */F*_*380*_ fluorescence ratio.

### Statistical analysis

Nonparametric Kruskal-Wallis or Mann-Whitney tests were done for the comparisons between groups or between two different experimental treatments respectively. Analyses were carried out with the GraphPad Prism version 5.01 software (GraphPad Software, San Diego, CA, USA). The data are presented as boxplots showing the 25^th^ and 75^th^ percentiles along with the median. The upper and lower bars show the maximum and minimum values. Probability (p) <0.05 was considered to be significant. For the statistical tests, N represents the size of the samples with data collected from cells isolated from different animals. When cited in the text, the data are expressed as mean ± SEM.

## Results

### Effect of hypotonic shock on transepithelial current

**Figure 1:**
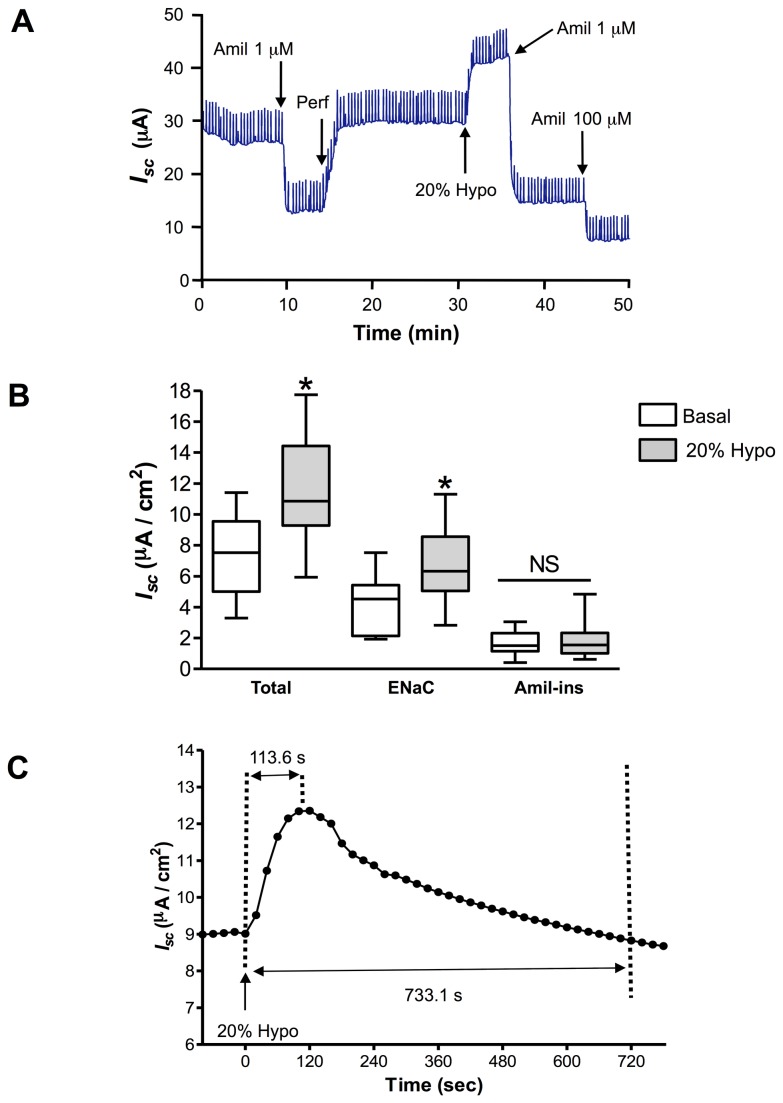
Modulation of *I*_*sc*_ after a 20% hypotonic shock in alveolar epithelial cells. A typical *I*_*sc*_ tracing is depicted in **A**. ENaC current was determined after adding 1 µM amiloride (Amil 1 µM) on the apical side. After apical medium perfusion (Perf) to wash away amiloride, the cells were subjected to bilateral 20% hypotonic shock (20% Hypo). Transepithelial resistance (R_te_) was monitored throughout the experiment by applying a 1-mV pulse every 10 s (seen as spikes in the *I*_*sc*_ trace). ENaC and amiloride-insensitive currents were then tested by adding 1 µM amiloride (Amil 1 µM) and 100 µM amiloride (Amil 100 µM) respectively. Total transepithelial current (Total), 1µM amiloride-sensitive ENaC current (ENaC) and the 100 µM amiloride–insensitive current (Amil-ins) are depicted in **B** for the untreated control condition (Basal) and after 20% hypotonic shock (20% Hypo). N≥10, *p<0.05 by Mann-Whitney Test between 20% Hypo and Basal. NS: non significant. Mean *I*_*sc*_ from different experiments shows the transient nature of the acute rise in the transepithelial current after 20% hypotonic shock (**C**). Mean time to reach maximum current is depicted on top (113.6 ± 7 s; N=17). The time to return to basal values is depicted at the bottom (733.1 ± 120 s; N≥5).

The consequence of bilateral 20% hypotonic shock on total and amiloride-sensitive *I*_*sc*_ generated by rat alveolar epithelial cells was investigated by Ussing chamber recording (Figure 1A). ENaC current sensitive to 1 µM amiloride was the main transepithelial current (60%) detected in alveolar epithelial cells (Figure 1A and 1B). Decreasing physiological buffer tonicity by 20% had an immediate and acute effect, raising the total transepithelial current (Total) by 52% from 7.52 ± 0.46 µA/cm^2^ to 11.43 ± 0.65 µA/cm^2^ (p<0.05; Figure 1B). This increase was mainly secondary to a 67% elevation of ENaC current (sensitive to 1 µM amiloride), from 4.14 ± 0.64 µA/cm^2^ to 6.92 ± 0.48 µA/cm^2^ (p<0.05; Figure 1B). When 100 µM amiloride was applied to inhibit NSC, a further 16% decrease was apparent (Figure 1A), demonstrating that NSC with low sensitivity to amiloride, made a smaller contribution than ENaC to total *I*_*sc*_ in these cells. The rise elicited by 20% hypotonic shock on the transepithelial current was transient, peaking at 113.6 ± 7 s (Figure 1C). The current was back to baseline value after ~12 min (733.1 ± 120 s). For this reason, we studied peak current after hypotonic treatment.

### Contribution of ENaC and K^+^ currents in acute modulation of transepithelial current after 20% hypotonic shock

**Figure 2:**
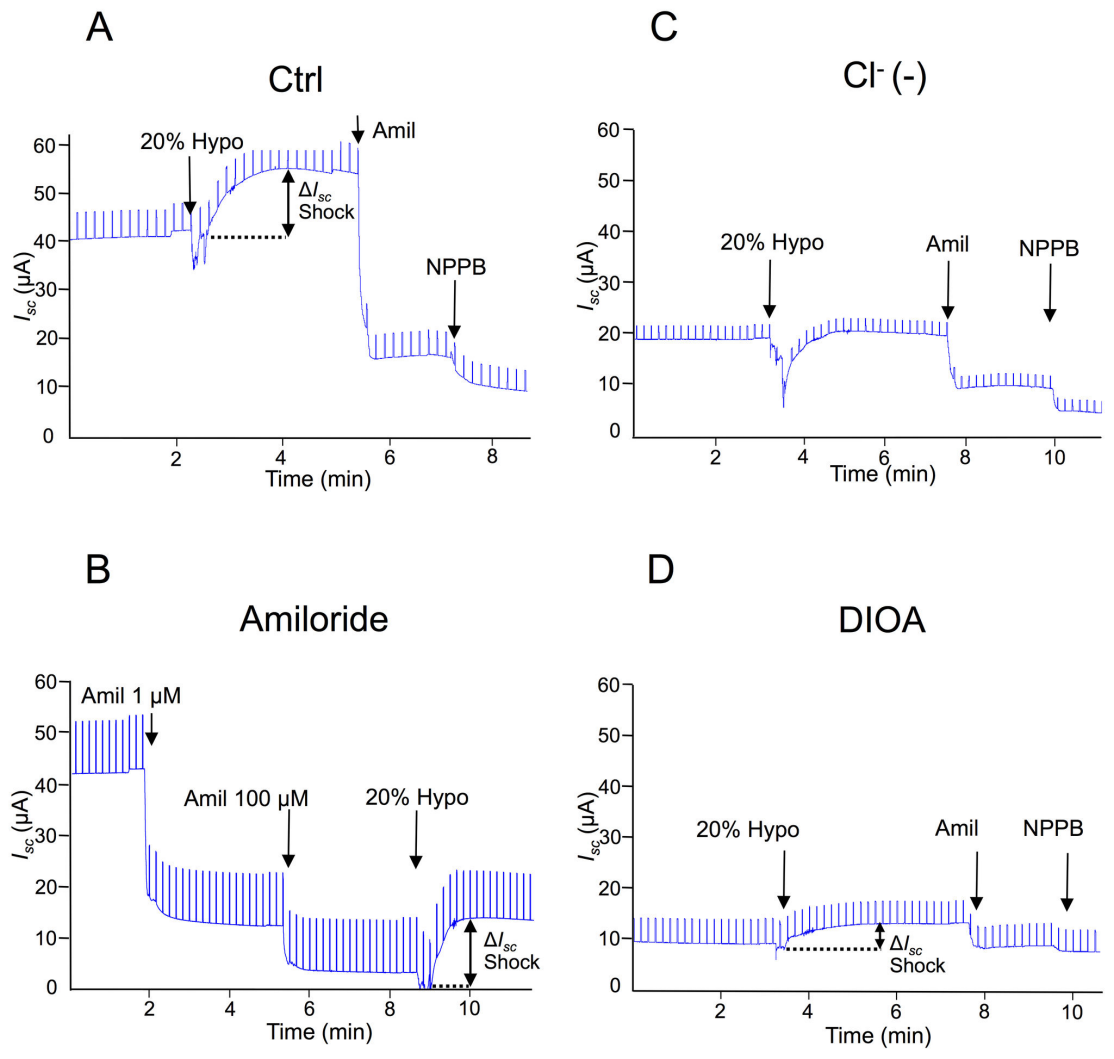
Impact of 20% hypotonic shock on the Ussing *I*_*sc*_ recording. Typical *I*_*sc*_ tracings are depicted for current responses to 20% hypotonic shock (20% Hypo) in the basal condition (Ctrl) **A**, after 100 µM amiloride pre-treatment (Amiloride) **B**, in reduced Cl^-^ buffer (9mM NaCl; 150 mM Na gluconate) (Cl^-^(-)) **C** or after 100 µM basolateral DIOA treatment **D**. Arrows show the start of hypotonic shock (20% Hypo) and the apical addition of 10 µM amiloride (Amil), 1 µM amiloride (Amil 1), 100 µM amiloride (Amil 100) or 100 µM NPPB. A double-headed arrow shows the current rise elicited by hypotonic shock (Δ *I*_*sc*_ Shock).

**Figure 3:**
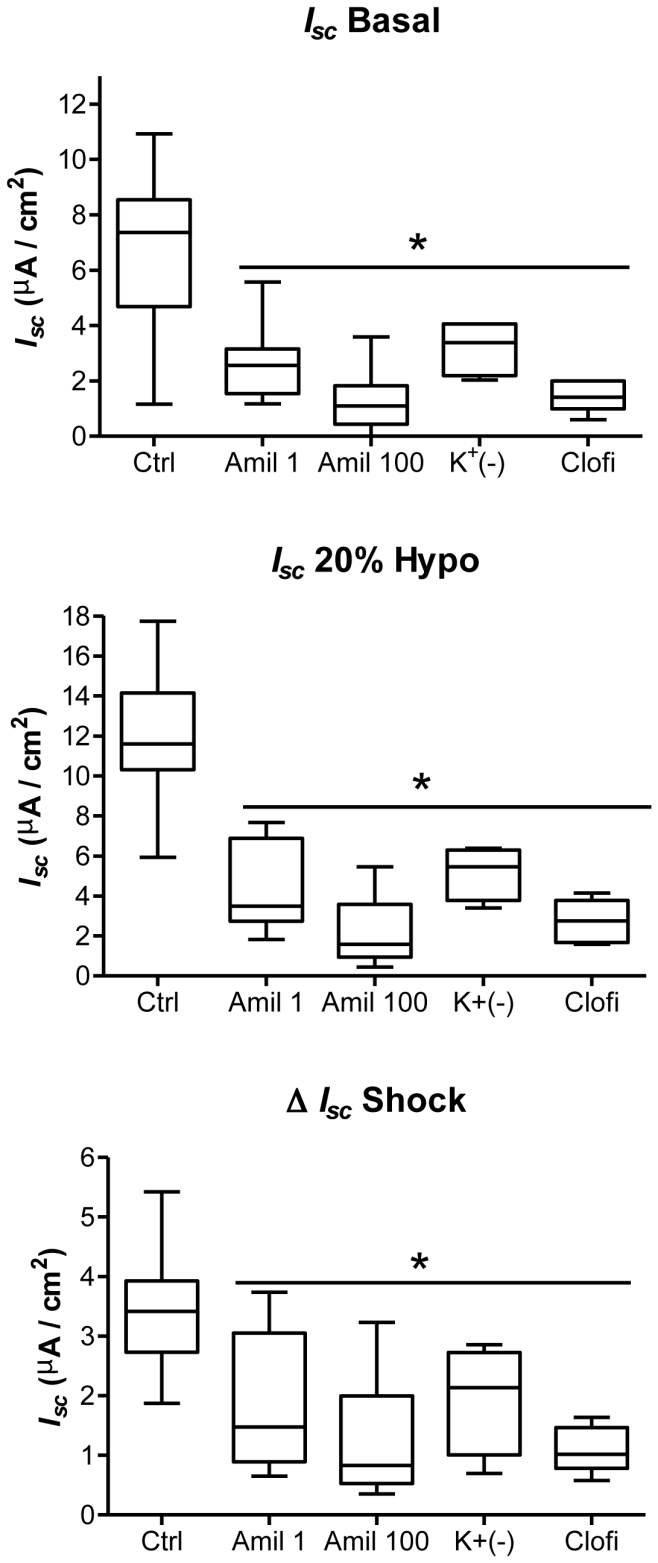
Role of Na^+^ and K^+^ currents in basal and hypotonic-induced transepithelial current. Basal transepithelial current (*I*_*sc*_ Basal), hypotonic-induced current (*I*_*sc*_ 20% Hypo) and the current rise elicited by hypotonic shock (Δ *I*_*sc*_ Shock) are depicted in the basal condition (Ctrl), potassium-free condition (K^+^(-)) or after pretreatment with 1µM amiloride (Amil 1), 100 µM amiloride (Amil 100) or 100 µM basolateral clofilium (Clofi). In experiments where the cells were pre-treated with an inhibitor that impacted on *I*_*sc*_, hypotonic shock was induced when the current was stabilized. For amiloride the current was stable after 5 min. In potassium-free condition (K^+^(-)) and clofilium pre-treatment, a longer incubation was needed (~30 min). N≥4, *p<0.05 by Mann-Whitney Test compared to untreated controls.

Hypotonic shock has been shown to modulate Na^+^ transport in kidney epithelial cells [25,27]. For this reason, we first tested the consequence of 1 µM (Amil 1) and 100 µM amiloride (Amil 100) pretreatment on basal and 20% hypotonic-induced transepithelial current. Typical Ussing chamber recordings for some of these experiments appear in Figure 2 with *I*_*sc*_ quantification reported in Figure 3. In control (Ctrl) cells, 20% hypotonic shock increased *I*_*sc*_ from 8.24 ± 0.34 µA/cm^2^ (*I*_*sc*_ Basal) to 11.90 ± 0.45 µA/cm^2^ (*I*_*sc*_ 20% Hypo; Figures 2A & 3). The current rise elicited by hypotonic shock (Δ *I*_*sc*_ Shock), depicted by the double-headed arrow in Figure 2, is also presented in Figure 3. Inhibition of ENaC with 1 µM amiloride (Amil 1) and of NSC with 100 µM amiloride (Amil 100) prior to hypotonic shock greatly reduced (p<0.05) basal and hypotonic-induced transepithelial current as well as Δ *I*_*sc*_ Shock (p<0.05) compared to untreated Ctrl cells (Figures 2B & 3). However, amiloride pre-treatment could not completely abolish the acute current rise after hypotonic shock (Figure 3). These results show that ENaC and NSC are important for the current rise after hypotonic shock. As Na^+^, K^+^-ATPase and K^+^ channels are crucial for the generation and modulation of transepithelial Na^+^ current, we tested the contribution of K^+^ to the current rise after hypotonic shock. Basal current decreased rapidly in buffer devoid of K^+^ (K^+^(-)) (p<0.05) (Figure 3). As with amiloride pretreatment, the current rise after 20% hypotonic shock was reduced but not abolished by modulation of K^+^ transport (Figure 3). Inhibition of KvLQT1, the main K^+^ channel detected in alveolar epithelial cells [35,36] with Clofilium tosylate (Clofi, 100 µM), had a similar impact as (K^+^(-)) buffer on basal and hypotonic-induced currents. These currents decreased significantly in comparison to untreated cells (p<0.05) (Figure 3). Although the treatment could not completely abolish the current rise after hypotonic shock, it completely inhibited the gradual decrease of current back to its basal value (data not included).

### Implication of Cl^-^ channels and KCC in basal and hypotonic-induced transepithelial current

**Figure 4:**
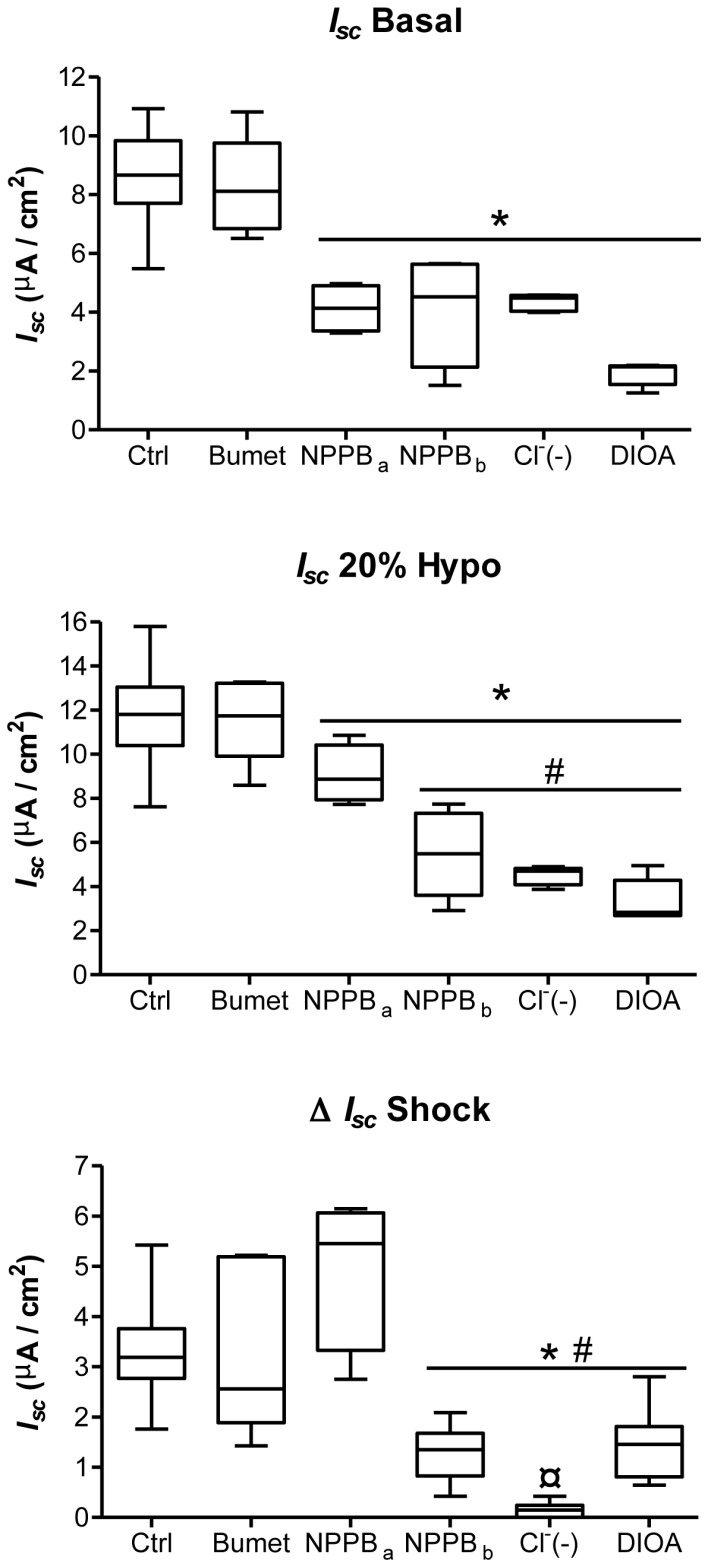
Implication of Cl^-^ channels and KCC in basal and hypotonic-induced transepithelial current. Basal transepithelial current (*I*_*sc*_ Basal), hypotonic-induced current (*I*_*sc*_ 20% Hypo) and the current rise elicited by hypotonic shock (Δ *I*_*sc*_ Shock) are depicted in the basal conditions (Ctrl), after treatment with 100 µM basolateral bumetanide (Bumet), 100 µM apical (NPPB_a_) or basolateral (NPPB_b_) NPPB, in bilateral Cl^-^ reduced buffer (Cl^-^(-)) or 100 µM basolateral DIOA. In experiments where the cells were pre-treated with an inhibitor that impacted on *I*_*sc*_, hypotonic shock was induced when the current was stabilized. For apical and basolateral NPPB, a 5 to 10 min incubation was needed while the current stabilized after ^~^30 min for DIOA. Pre-treatment with bumetanide from 10 min to 30 min did not have an impact on *I*_*sc*_ Basal. N≥4, *p<0.05 by Mann-Whitney Test compared to untreated controls. # p<0.05 by Mann-Whitney Test compared to NPPB_a_, ¤ p<0.05 by Mann-Whitney Test compared to NPPB_b_ or DIOA.

**Figure 5:**
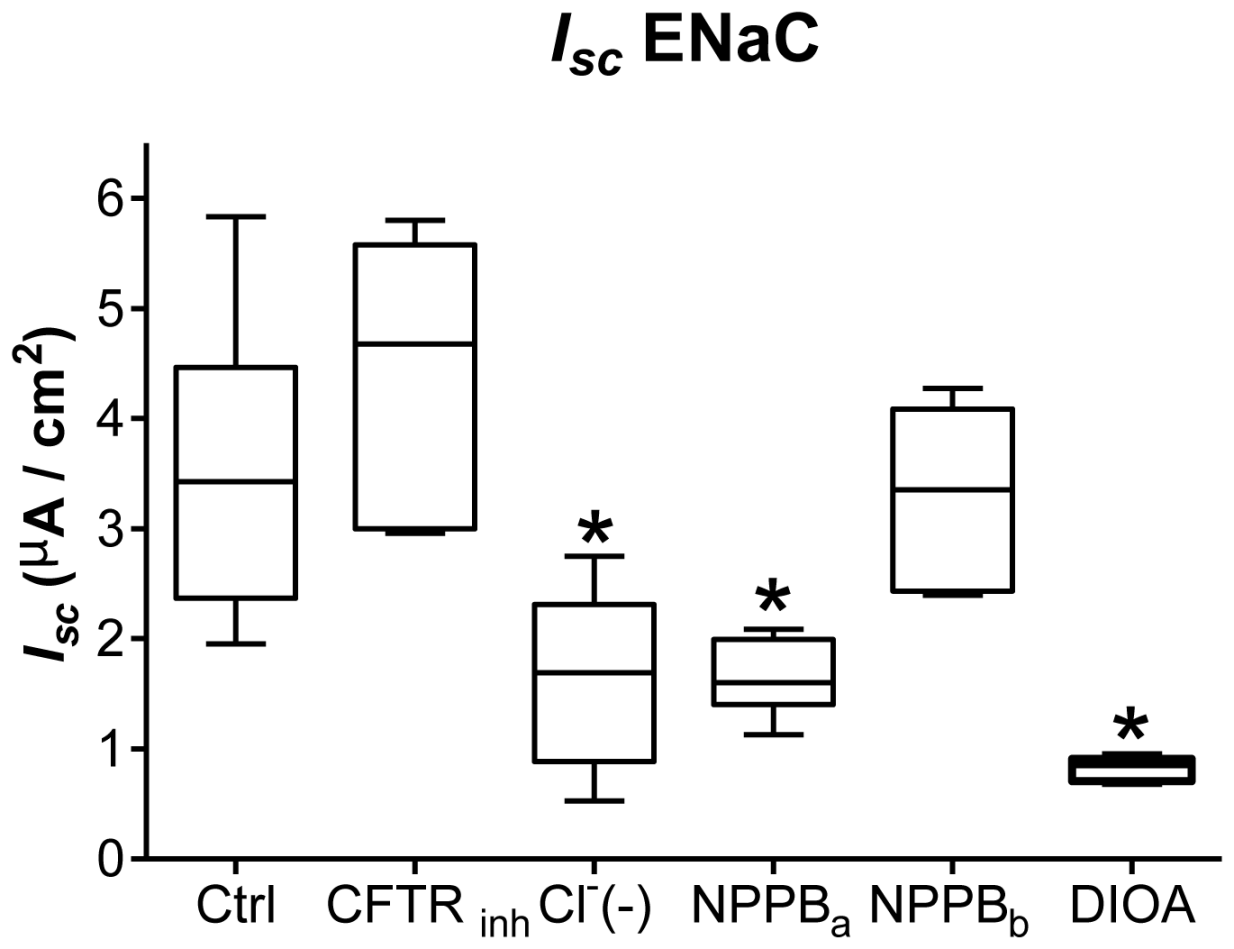
Role of apical Cl^-^ channels and KCC co-transport in ENaC current magnitude. ENaC current values were determined after pretreatments with 20 µM CFTR inh-172 (CFTR inh), reduced Cl^-^ buffer (9mM NaCl; 150 mM Na gluconate) (Cl^-^(-)), 100 µM apical NPPB (NPPB_a_), 100 µM basolateral NPPB (NPPB_b_), or 100 µM basolateral DIOA. In experiments where the cells were pre-treated with an inhibitor that impacted on *I*_*sc*_, hypotonic shock was induced when the current was stabilized. For apical and basolateral NPPB, a 5 to 10 min incubation was needed while the current stabilized after ^~^30 min for DIOA. Pre-treatment with CFTR inh-172 and NPPB_b_ did not have an impact on *I*_*sc*_ ENaC. N≥5, *p<0.05 by Mann-Whitney Test compared to untreated controls (Ctrl).

**Figure 6:**
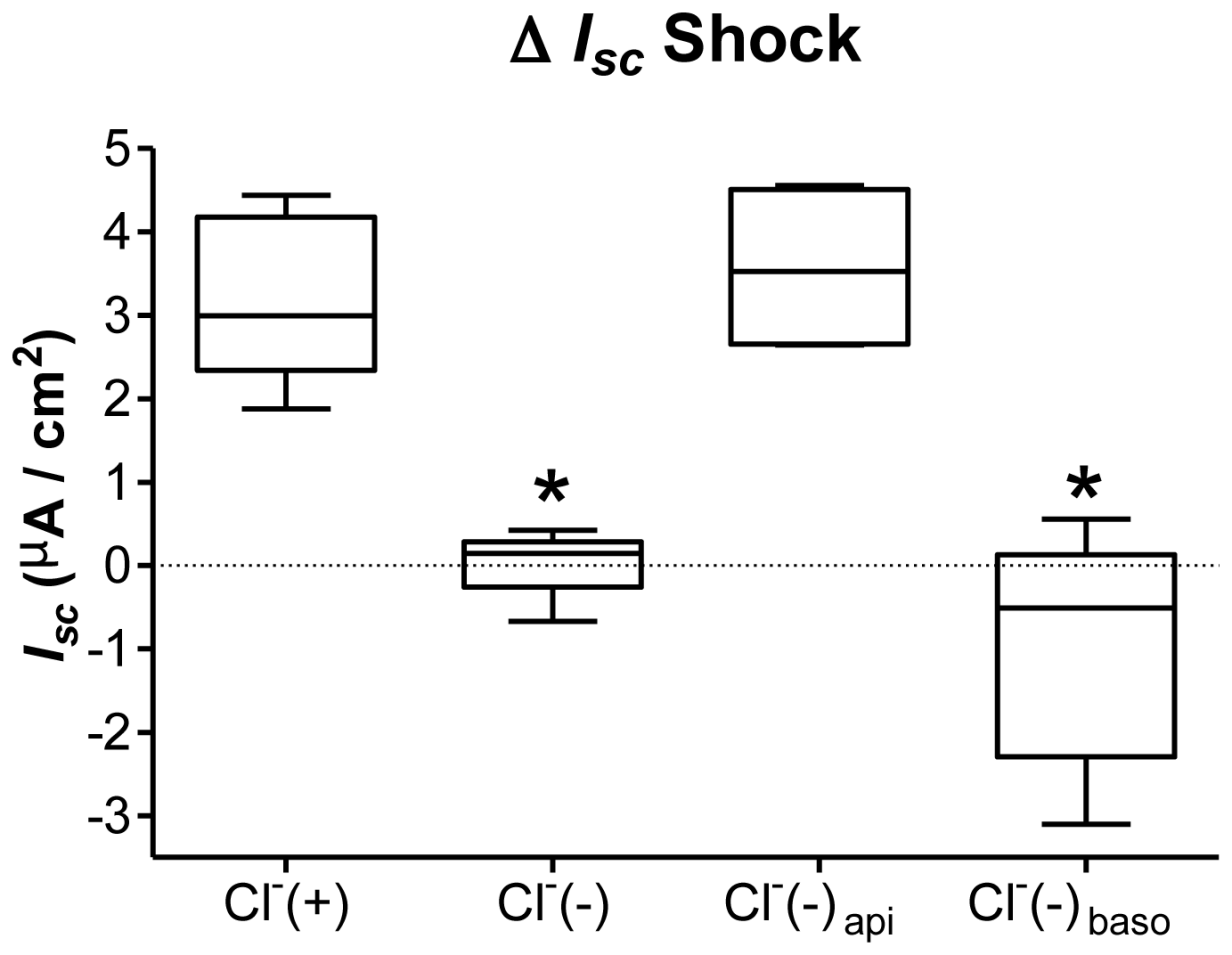
Impact of apical or basolateral Cl^-^ (-) gradients on the current rise elicited by 20% hypotonic shock. The current rise elicited by hypotonic shock (Δ *I*_*sc*_ Shock) was tested in the basal condition (Cl^-^(+)), with bilateral exposure to a buffer of reduced Cl^-^ concentration (9 mM NaCl; 150 mM Na gluconate) (Cl^-^(-)), or by adding this buffer at the apical side (Cl^-^(-) api) or basolateral side (Cl^-^(-) baso) only. N≥4, *p<0.05 by Mann-Whitney Test compared to Cl^-^(+).

Cytosolic Cl^-^ has been shown to be important for ENaC modulation after hypotonic shock [26,37]. For this reason, several strategies were tested to determine if Cl^-^ transport could be involved in the current rise after hypotonic shock. Basal (*I*_*sc*_ Basal) and hypotonic-induced currents (*I*_*sc*_ 20% Hypo) were recorded after pretreatment of alveolar epithelial cells with 100 µM basolateral bumetanide (Bumet), a Na, K,2Cl co-transporter inhibitor, 100 µM apical (NPPB_a_) or basolateral (NPPB_b_) NPPB, a broad Cl^-^ channel inhibitor, 100 µM basolateral DIOA, a K^+^/Cl^-^co-transporter (KCC) inhibitor or a reduced Cl^-^ buffer (Cl^-^ (-)). Typical Ussing recording in some of these experiments are depicted in Figure 2C,D, and *I*_*sc*_ quantifications appear in Figure 4. Bumetanide had no impact on basal and hypotonic-induced currents (Figure 4). NPPB_a_, NPPB_b_ low chloride (Cl^-^ (-)) and DIOA significantly reduced (p<0.05) *I*_*sc*_ Basal (Figure 4), indicating that Cl^-^ channels and co-transporters play important roles in basal transepithelial current. To further test the co-dependence of Cl^-^ in ENaC current generation, we investigated the impact of different inhibitors of Cl^-^ channels and co-transporters on ENaC current in the basal condition (Figure 5). While pretreatment with the CFTR inhibitor CFTR inh-172 (inh CFTR) and basolateral NPPB had no impact, a low Cl^-^ buffer (Cl^-^(-)), NPPB_a_ and DIOA significantly reduced the amplitude of ENaC current (*I*_*sc*_ ENaC) (Figure 5). NPPB_a_, NPPB_b_, DIOA and low Cl^-^ buffer also significantly diminished (p<0.05) 20% hypotonic-induced current compared to control cells (*I*_*sc*_ 20% Hypo; Figure 4). However, only NPPB_b_, low Cl^-^ buffer and DIOA significantly decreased the amplitude of the current rise induced by hypotonic shock (Δ *I*_*sc*_ Shock, Figure 4). Bilateral low Cl^-^ buffer (Cl^-^(-)) (Figure 2C) was the only condition that completely abolished any current rise after 20% hypotonic shock (¤ p<0.05 compared to NPPB_b_ or DIOA; Figure 4). To further test the nature of the membrane involved in hypotonic Cl^-^ transport, the current rise elicited by hypotonic shock (Δ *I*_*sc*_ Shock) was examined in the control condition (Cl^-^(+)), in the presence of a bilateral reduced Cl^-^ buffer (Cl^-^(-)), and in the presence of either reduced apical (Cl^-^(-) api) or basolateral (Cl^-^(-) baso) Cl^-^ concentration (Figure 6). While decreased Cl^-^ concentration on the apical side had no impact on the amplitude of Δ *I*_*sc*_ Shock, reduced Cl^-^ concentration on the basolateral side or on both sides completely abolished the hypotonic-induced *I*_*sc*_ rise (Figure 6).

### Modulation of calcium by hypotonic shock in alveolar epithelial cells

**Figure 7:**
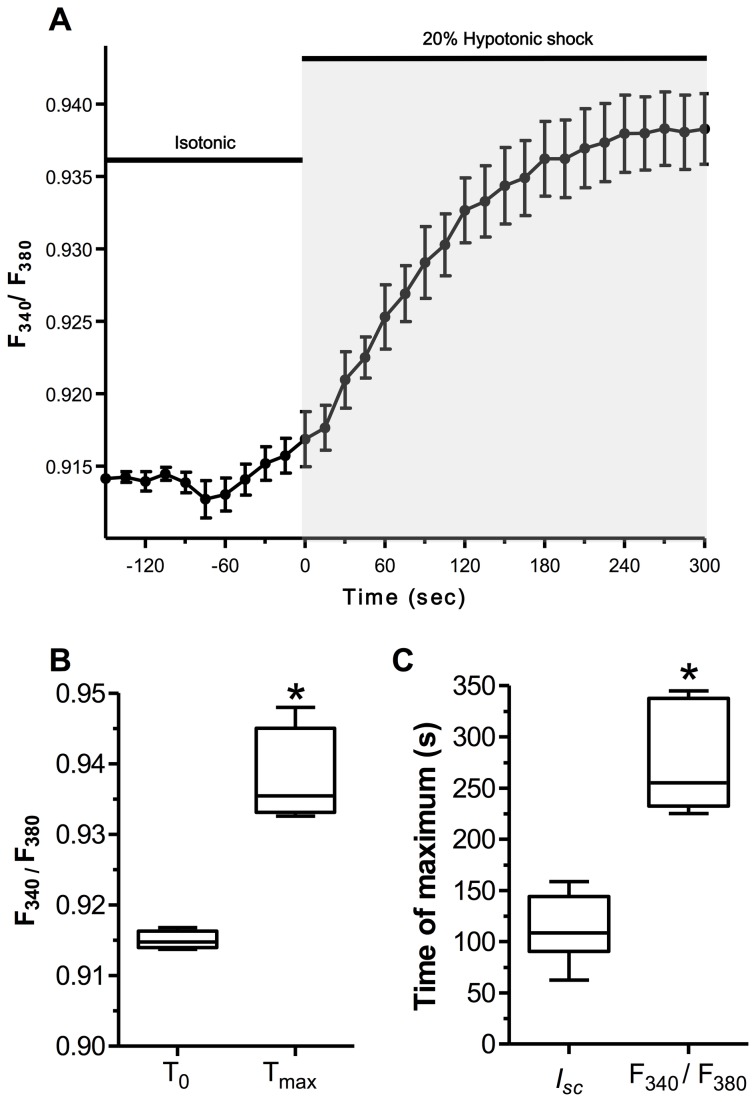
Kinetic of [Ca^2+^]_i_ concentration rise after 20% hypotonic shock in alveolar epithelial cells. F_340_/F_380_ ratio kinetic of the [Ca^2+^]_i_ rise detected by Fura-2 Ca^2+^ fluorescence after 20% hypotonic shock in alveolar epithelial cells is depicted in **A**. Hypotonic shock induced an immediate increment of intracellular [Ca^2+^]_i_, reaching maximum after 266.3 ± 27 s (T_max_) compared to the basal condition (T_0_). The F_340_/F_380_ ratio rose significantly between T_0_ and T_max_ (N=4, *p<0.05 by Mann-Whitney Test) (**B**). The time to reach maximum *I*_*sc*_ and maximum [Ca^2+^]_i_ was significantly different (N=17 for *I*_*sc*_, N=5 for F_340_/F_380_, *p<0.05 by Mann-Whitney Test) (**C**).

**Figure 8:**
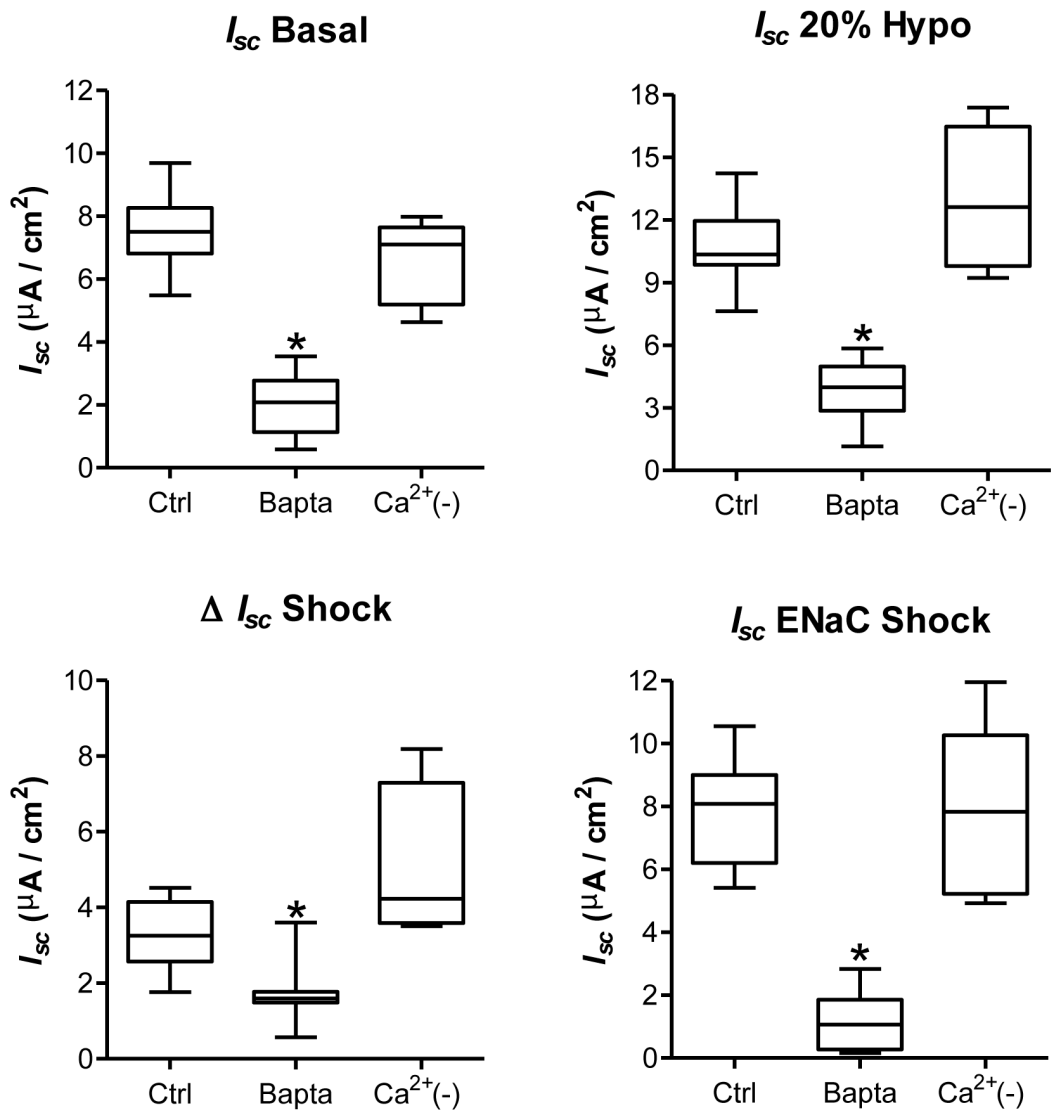
Implication of Ca^2+^ in basal and hypotonic-induced transepithelial current. Basal transepithelial current (*I*_*sc*_ Basal), hypotonic-induced current (*I*_*sc*_ 20% Hypo), the current rise elicited by hypotonic shock (Δ *I*_*sc*_ Shock) and the amplitude of ENaC current recorded after hypotonic shock (*I*_*sc*_ ENaC Shock) are depicted in basal condition (Ctrl), after treatment for 20 min with 50 µM BAPTA-AM (Bapta) or in a physiological buffer devoid of Ca^2+^ (Ca^2+^(-)). N≥5, *p<0.05 by Mann-Whitney Test compared to untreated controls.

Hypotonic shock has been shown to induce rapid intracellular Ca^2+^ ([Ca^2+^]_i_) elevation in A549 cells [29,30]. For this reason, Fura-2 fluorescence was monitored after 20% hypotonic shock to determine if the treatment was affecting cytosolic Ca^2+^ levels [Ca^2+^]_i_ in alveolar epithelial cells (Figure 7A). As with *I*_*sc*_, 20% hypotonic shock induced an immediate rise in [Ca^2+^]_i,_ which was significant between T_0_ and the time of maximum value (T_max_) (p<0.05; Figure 7B). The kinetics of change brought by hypotonic shock were, however, different between transepithelial current and [Ca^2+^]_i_. Peak Ca^2+^ rise post-hypotonic shock was reached after a longer time (266 ± 27 s) compared to the time for reaching maximum current (p<0.05; Figure 7C). To determine how intracellular and extracellular Ca^2+^ was important for modulation of the basal and hypotonic shock current, *I*_*sc*_ measurements were repeated following 50 µM BAPTA-AM pre-treatment (BAPTA) or in absence of Ca^2+^ (Ca^2+^(-)) in the physiological buffer (Figure 8). While the absence of extracellular Ca^2+^ had no consequences on the basal (*I*_*sc*_ Basal) and hypotonic-induced currents (*I*_*sc*_ 20% Hypo), these currents were significantly decreased (p<0.05) after BAPTA pre-treatment (Figure 8). In addition, BAPTA decreased the hypotonic-induced transepithelial current (Δ *I*_*sc*_ Shock) and almost abolished the ENaC hypotonic shock current (*I*_*sc*_ ENaC shock).

### Inhibition of calmodulin kinase by W7 decreases the hypotonic shock current rise

**Figure 9:**
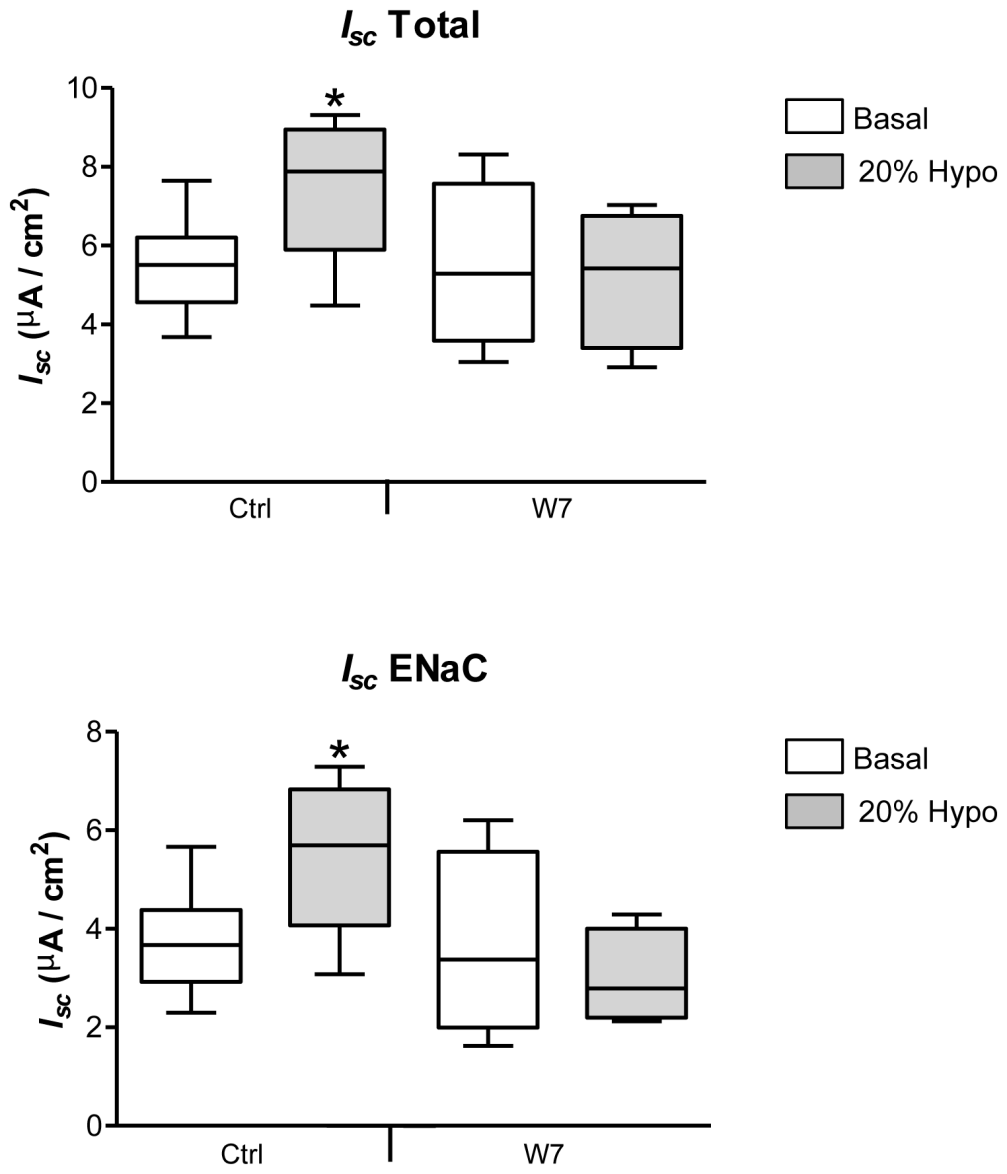
Inhibition of calmodulin kinase by W7 decreases the hypotonic shock current rise. Alveolar epithelial cells were pretreated for 20 min with 25 µM W7, a Ca^2+^/calmodulin antagonist, before *I*_*sc*_ recording in Ussing chamber. Total (*I*_*sc*_ Total) and ENaC currents were evaluated in basal condition (Basal; white) or after 20% hypotonic shock (20% Hypo; gray). W7 pretreatment inhibited the total and ENaC *I*_*sc*_ rise after 20% hypotonic shock. N≥4, *p<0.05 by Mann-Whitney Test compared to Basal.

Calmodulin has been shown to modulate the rise in ENaC-mediated current after hypotonic shock in A6 renal epithelial cells [27]. We tested if W7 (25 µM; N-(6-aminohexyl)-5-chloro-1-naphtalene-sulfonamide), a Ca^2+^/calmodulin antagonist, could influence the current rise elicited by hypotonic shock in alveolar epithelial cells. While 25 µM W7 pretreatment had no impact on basal and ENaC transepithelial current, it inhibited total and ENaC transepithelial current increment after 20% hypotonic shock (Figure 9). This result indicates that Ca^2+^ and calmodulin signaling plays a role in the current rise induced by hypotonic shock in alveolar epithelial cells.

### Hypotonic shock evokes ATP release

**Figure 10:**
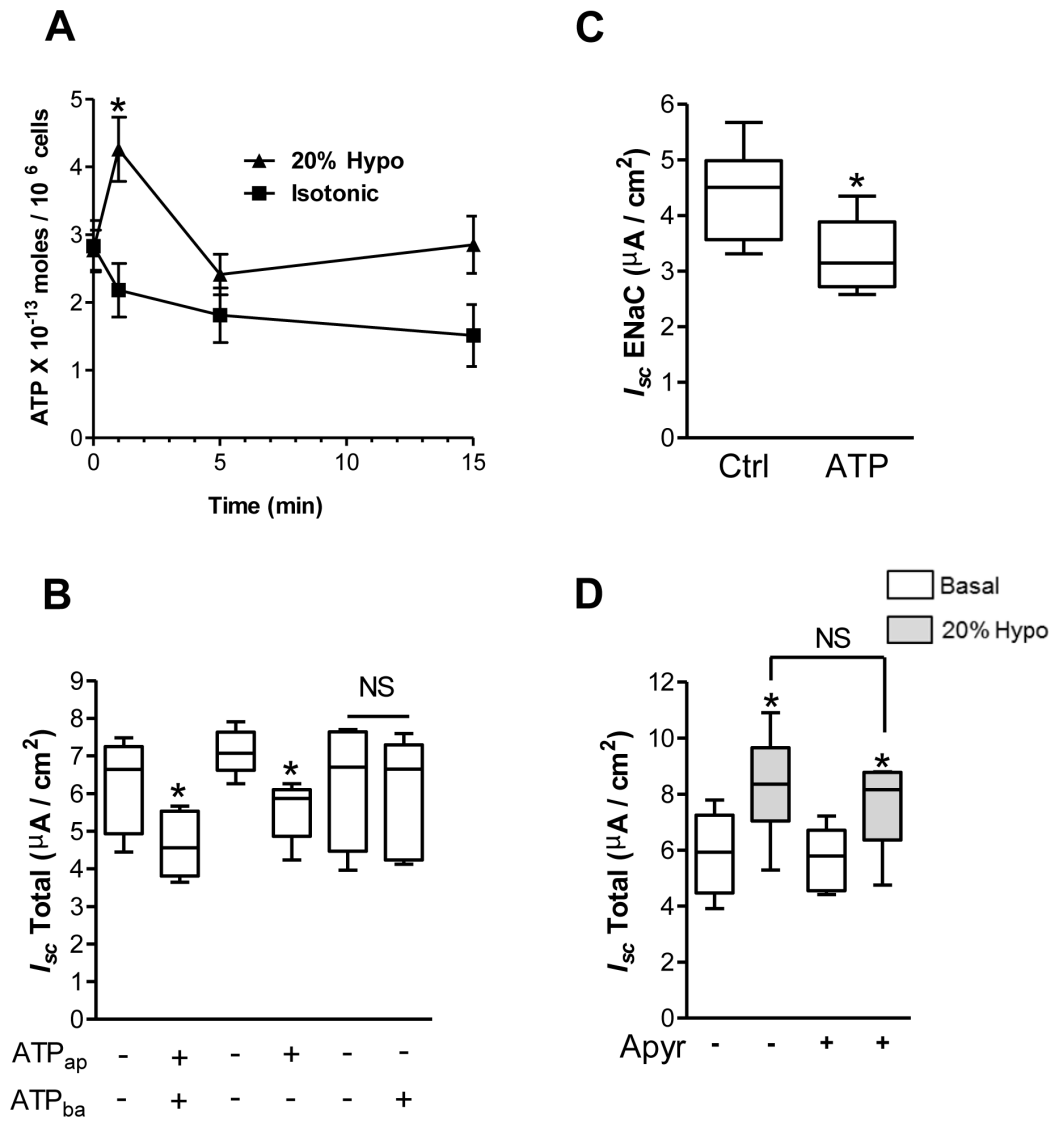
ATP secretion after hypotonic shock and acute effect on *I*_*sc*_ generated by alveolar epithelial cells. The time-course of 20% hypotonic shock (20% hypo) in apical ATP accumulation is depicted in A. At T_0_, the same volume of liquid was added to the apical and basolateral sides of the cell monolayers to achieve 20% hypotonic shock (▲) with H_2_O or an equivalent volume of physiological buffer (■) for the isotonic condition. Aliquots of apical medium were sampled at different time points, and ATP was measured by luciferin/luciferase assay. N≥7, *p<0.05 by Mann-Whitney Test between 20% Hypo at 1 min and time 0. Apical (ATP_ap_) or bilateral, but not basolateral (ATP_ba_) addition of ATP, decreased total transepithelial current generated by alveolar epithelial cells (**B**). N≥5, *p<0.05 by Mann-Whitney Test between apical or bilateral addition of ATP and untreated cells. NS, non significant. Bilateral addition of ATP decreased ENaC (1 µM amiloride-sensitive) current (**C**). N=6, *p<0.05 by Mann-Whitney Test between ATP and untreated controls (Ctrl). To rule out a role of ATP in hypotonic shock modulation of currents, the cells were pretreated with the ATP scavenger apyrase (10 U/ml; Apyr) for 2 min before challenging the monolayers with 20% hypotonic shock (**D**). Apyrase treatment had no significant effect on the *I*_*sc*_ increase induced by hypotonic shock. N≥4, NS: non significant by Mann-Whitney Test between 20% Hypo in presence of absence of Apyr. *p<0.05 by Mann-Whitney Test between basal (white) or 20% hypo (grey) treated cells.

We reported previously that hypotonic shock induces ATP release from several cell types, including lung epithelial cells [29,38] and that purinergic signaling is implicated in the [Ca^2+^]_i_ rise [29]. Since ATP may contribute to Na^+^ transport modulation by paracrine/autocrine action on purinergic receptors, apical and basolateral ATP concentration was tested by luciferase assay after 20% hypotonic shock in alveolar epithelial cells. Hypotonic shock induced transient but significant ATP accumulation (p<0.05) on the apical side of the monolayer peaking after 1 min (1.77 X 10^-13^ moles/10^6^ cells above the baseline), followed by a rapid decrease within 5 min (Figure 10A). ATP accumulation was not detected on the basolateral side of the monolayers (data not reported).

### Effect of extracellular ATP on I_sc_

ATP has been shown to modulate ENaC and transepithelial ionic transport in different epithelial cell types [39–41]. We investigated if direct addition of exogenous ATP, in the absence of hypotonic shock, could modulate Na^+^ transport. Application of 100 µM ATP on both sides of the monolayer reduced total *I*_*sc*_ by 25% from 6.25 ± 0.51 to 4.68 ± 0.36 µA/cm^2^ (Figure 10B). A similar *I*_*sc*_ decrease, from 7.11 ± 0.27 to 5.56 ± 0.35 µA/cm^2^, was seen when ATP was applied at the apical side (Figure 10B). ATP added at the basolateral side had no effect (Figure 10B). Decline of total *I*_*sc*_ by bilateral ATP was mainly related to amiloride-sensitive current reduction since the treatment significantly decreased ENaC current from 4.27 ± 0.25 to 3.36 ± 0.310 µA/cm^2^ (Figure 10C). We observed significant ecto-ATPase activity at the surface of alveolar epithelial cells. When exogenous ATP was added to these cells, a rapid break down occurred with a maximal rate of 2.14 nM/sec in cells grown on filters. We, therefore, tested if adenosine, the end product of ATP degradation, could modulate the current generated by these cells. Apical and basolateral addition of 100 µM adenosine had no acute impact on the current (not shown).

### Effect of ATP scavenger on the transepithelial current induced by hypotonic shock

Exogenous ATP and hypotonic shock have opposite effects on transepithelial current generated by alveolar epithelial cells. To further test that ATP was not involved in current modulation by hypotonic shock, the cell monolayers were pretreated for 2 min with 10 U/ml apyrase, an ATP scavenger (Apyr), before challenging them with 20% hypotonic shock. Apyrase treatment could not prevent the current rise elicited by hypotonic shock (from 5.01 ± 0.46 µA/cm^2^ to 8.19 ± 0.45 µA/cm^2^), ruling out a role of ATP in this process (Figure 10D).

## Discussion

Transepithelial Na^+^ transport by alveolar epithelial cells is an important process that maintains an optimal volume of liquid lining the respiratory epithelium. In addition to ENaC and Na^+^/K^+^-ATPase activity at the apical and basolateral sides of cells, recent works have shown that Cl^-^ and K^+^ channels and transporters could be important in modulating Na^+^ transport in these cells [21,23]. Since different Cl^-^ and K^+^ channels could be activated after hypotonic shock, we tested how mild hypotonic shock (20%) could modulate total and amiloride-sensitive current in alveolar epithelial cells. We noted an acute but transient rise in total and amiloride-sensitive currents induced by 20% hypotonic shock. Moreover, we found that Cl^-^ transport is important for the basal transepithelial current generated by alveolar epithelial cells and for the current rise induced by hypotonic shock.

Challenging alveolar epithelial cells with 20% hypotonic shock induced an acute and immediate transepithelial current rise where total and ENaC transepithelial currents were increased by 52% and 67%, respectively. A similar observation has been reported in A6 kidney epithelial cells [25,27,42,43]. There are significant differences, however, in modalities of the hypotonic shock response in kidney cells and alveolar epithelial cells. When hypotonic shock induced an adaptive response in A6 cells, which leads to a sustained gradual increase in ENaC and amiloride-sensitive current over time [28], the rapid current rise in alveolar epithelial cells is transient and returns gradually to basal values after ~12 min. Clearly, the response of alveolar epithelial cells to hypotonic shock is different from that of kidney cells which have to adapt their ionic transport to the changing tonicity of the tubular environment. Some studies have reported changes in Rb^+^ uptake in response to different tonicity challenges in alveolar epithelial cells [44]. To the best of our knowledge, the present investigation is the first to describe the impact of hypotonicity on transepithelial current generated in rat alveolar epithelial cells.

Pre-treatment of cells with amiloride significantly decreased basal current (*I*_*sc*_ Basal) and the hypotonic-induced current (*I*_*sc*_ 20% Hypo), indicating that ENaC-mediated current is one of the major ionic transports in basal and hypotonic shock conditions. Interestingly, other treatments, known to decrease transepithelial Na^+^ current, such as inhibition of the KvLQT1 K^+^ channels with clofilium or K^+^(-) medium [35,36], had a similar impact as amiloride on basal, and hypotonic-induced current. The ENaC current response to hypotonic shock is different from that in *Xenopus laevis* oocytes where hypotonicity results in decreased amiloride-sensitive current [45,46]. In alveolar epithelial cells as in kidney cells, ENaC transport modulation during hypotonic shock is, therefore, different from that seen in *Xenopus* oocytes. This probably reflects the nature of different cell volume strategies, including the nature of channels and transporters activated by hypotonic shock in epithelial cells that express ENaC endogenously. In the present study, amiloride did not completely inhibit the increase in current after hypotonic shock (Δ *I*_*sc*_ shock) (Figure 3), indicating that, in addition to ENaC, other channels and transporters could be involved in the hypotonic-induced current rise. It is well known that Cl^-^ channels and K^+^/Cl^-^ co-transporters (KCC) are activated after hypotonic shock [24,47,48]. For this reason, different strategies were tested to investigate how Cl^-^ could be associated with basal and hypotonic transepithelial current responses.

While Bumet a Na^+^/K^+^/2Cl^-^ co-transporter inhibitor, had no impact on basal current, all treatments aimed at reducing Cl^-^ transport significantly decreased basal transepithelial current (Figure 4). The importance of Cl^-^ transport in amiloride-sensitive Na^+^ transport (ENaC current) was also evaluated. Pretreatment of cell monolayers with apical but not basolateral NPPB, a broad Cl^-^ channel inhibitor, greatly reduced ENaC current (Figure 5). Basolateral DIOA, a KCC inhibitor or Cl^-^(-) buffer also inhibited ENaC current. Although NPPB has been shown to decrease fluid clearance in the lungs [49], the results presented here are the first to disclose that Cl^-^ transport plays a significant role in the generation of ENaC transepithelial current in unstimulated alveolar epithelial cells. Nature of the apical NPPB-sensitive Cl^-^ channels that could be involved in basal Na^+^ current is not known so far. Patch clamping detected, large conductance Cl^-^ channels in alveolar epithelial cells from adult rats [50], and large conductance G protein-regulated Cl^-^ channels have been reported in freshly isolated alveolar epithelial cells from foetal guinea pig lungs [51]. CLC2 and CLC5 voltage-gated Cl^-^ channels have been found to be expressed in freshly isolated alveolar type I cells from rat lungs [52]. Basolateral Cl^-^ extrusion via KCC is essential in transcellular Cl^-^ transport [53]. Alveolar epithelial cells have been shown to express 2 isoforms of the K^+^/Cl^-^ co-transporter: KCC1 and KCC4 protein [53]. Data from the present study are the first to disclose that DIOA, a KCC inhibitor, has an impact on basal transepithelial current. It has been proposed that apical Cl^-^ channel activity could increase the driving force for Na^+^ because of the resulting membrane hyperpolarization [22,54] while K^+^/Cl^-^ co-transport, by recycling K^+^, could help to sustain Na^+^–K^+^-ATPase activity [53]. Studies of gastric cells have revealed that some KCC isoforms can co-immunoprecipitate with Na^+^–K^+^-ATPase [55] and up-regulate Na^+^–K^+^-ATPase activity [56]. The KCC/Na^+^-K^+^-ATPase relationship should be further investigated in alveolar epithelial cells.

It has been known for several years that β-agonists [54,57] or cAMP elevation by forskolin [58] increased Na^+^ transport in alveolar epithelial cells via apical Cl^-^ channel activation. CFTR is expressed in alveolar epithelial cells [52,59] and its activation is important for lung liquid clearance [49] and liquid transport by alveolar epithelial cells in culture [60]. The results presented here show that, in the absence of cAMP agonist, CFTR is not involved in basal ENaC current since CFTR inh 172 had no effect (Figure 5), confirming the observation that un-stimulated CFTR has no impact on fluid transport across alveolar epithelial cells [60].

**Figure 11:**
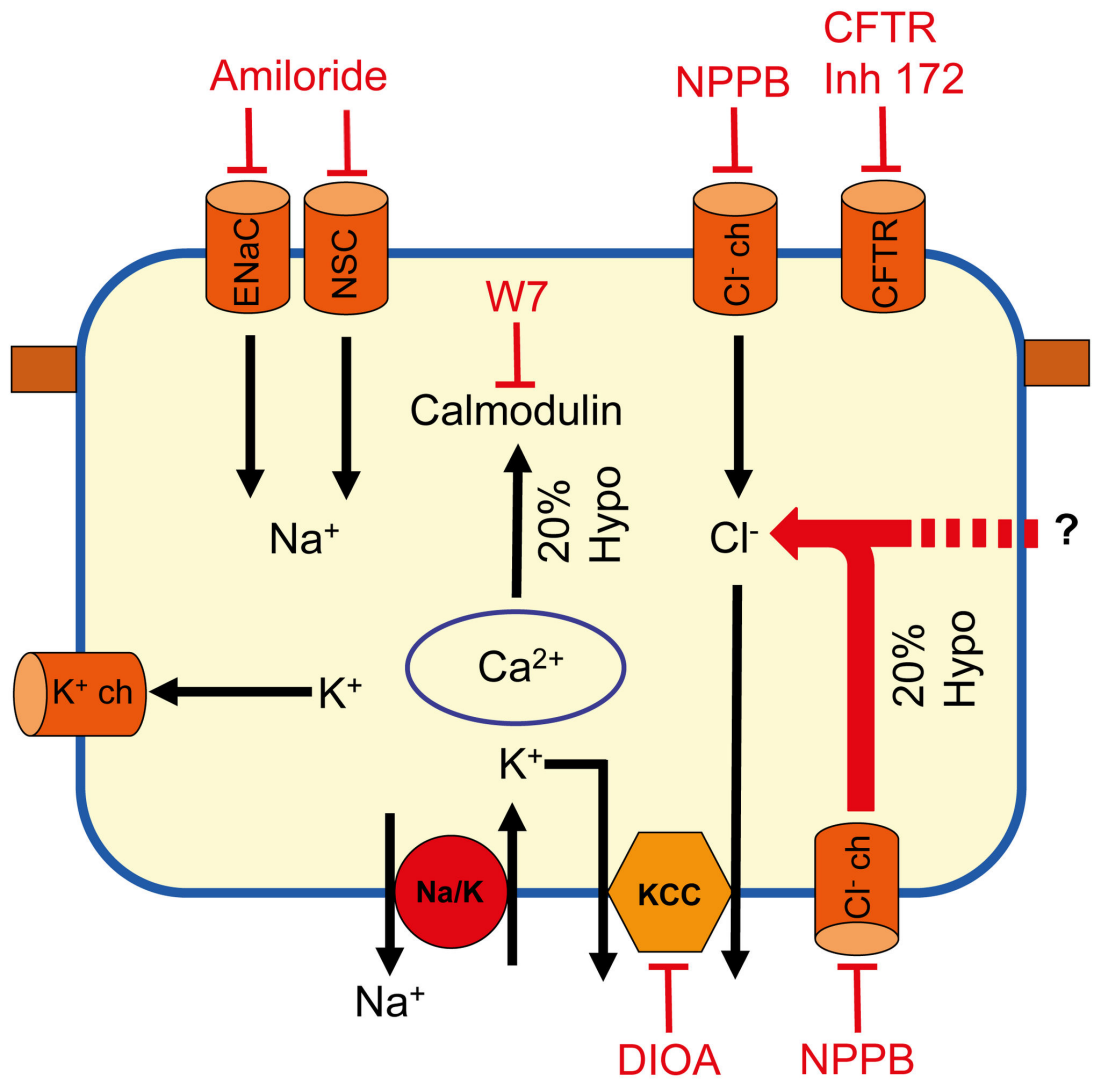
Channels and transporters involved in ionic transport in basal and hypotonic shock conditions. In the basal condition, a transcellular Cl^-^ transport via NPPB-sensitive apical channels (Cl^-^ ch) and basolateral K^+^/Cl^-^ co-transporter (KCC) generates membrane potential that is optimal for Na^+^ transport via amiloride-sensitive ENaC and NSC. Although alveolar epithelial cells express CFTR [59], in the absence of a cAMP agonist, CFTR is not involved in basal Na^+^ transport. During a 20% hypotonic shock, Ca^2+^/calmodulin and a basolateral Cl^-^ Influx play a central role in the transient current rise elicited by hypotonicity. NPPB-sensitive and insensitive pathways are involved for this flux. Although basolateral K^+^ channels (K^+^ ch) are not involved in the current rise elicited during hypotonic shock, their inhibition blunts current normalization after hypotonic shock. →: Ionic flux; **T** : Inhibitor.

In addition to basal transepithelial and ENaC current, basolateral Cl^-^ plays an important role in the current rise after hypotonic shock (Δ *I*_*sc*_ Shock) since basolateral NPPB, DIOA and basolateral Cl^-^ replacement reduced Δ *I*_*sc*_ Shock (Figures 4, 6). Chloride substitution at the basal side was the only condition that inhibits completely the hypotonic current rise. As basolateral NPPB inhibited only in part the hypotonic current rise, it suggests that hypotonicity triggers basolateral NPPB-sensitive as well as insensitive Cl^-^ influx that have consequence on the transeptithelial current. This is similar to what has been reported in A6 cells where an NPPB-sensitive outwardly Cl^-^ channel was activated during hypotonic shock [47]. Several mechanisms could account for the chloride-dependent hypotonic current rise. A decrease in cytosolic chloride concentration [Cl^-^]_i_ has been shown to occur rapidly following hypotonic challenge in A6 cells [37,47]. Several studies report that a decrease in [Cl^-^] could increase NSC channel activity in foetal rat pneumocytes [61] and ENaC in M1 kidney cells [62]. As high cytosolic [63,64] and extracellular Cl^-^ [65] concentrations have been shown to inhibit ENaC, the acute rise of the transepithelial current in hypotonic condition could be explained in part by an activation of ENaC following a decrease in cytosolic and extracellular Cl^-^. In addition to ENaC activation, a basolateral outwardly Cl^-^ current activated by hypotonic challenge could also contribute to the current rise. Ano1/TMEM16a is a calcium activated chloride channel (CaCC) present at the apical membrane that is expressed in the lung [66,67] and has been shown to promote regulatory volume decrease following hypotonic shock [68]. Although Ano1/TMEM16a activation is calmodulin-dependent [69] and is known to be stimulated by calmodulin, the observation that apical NPPB could not inhibit the current rise elicited by hypotonic shock (Figure 4) precludes a role for this channel during hypotonic challenge in alveolar epithelial cells. Altogether, these data show that basal and hypotonic transepithelial current is the result of a complex interplay between Cl^-^, K^+^ and Na^+^ transport. A scheme of the channels and transporters involved in this process is depicted in Figure 11.

We reported previously that hypotonic shock induces rapid Ca^2+^ mobilization from intracellular stores in A549 cells [29–31]. For this reason, we tested how hypotonic shock modulates [Ca^2+^]_i_ in rat alveolar epithelial cells. As reported in Figure 7A, 20% hypotonic shock led to an immediate but gradual rise of [Ca^2+^]_i_ that reached a plateau after ~4.5 min. The response was different from that in A549 cells where hypotonic shock induced a strong but transient [Ca^2+^]_i_ spike from thapsigargin-sensitive stores, followed by a sustained plateau from thapsigargin-insensitive stores [29,30]. In rat alveolar epithelial cells, hypotonic shock led to a sustained plateau reminiscent of Ca^2+^ mobilization from thapsigargin-insensitive stores, without the rapid spike seen in A549 cells.

Is the [Ca^2+^]_i_ rise important for transepithelial current modulation by hypotonic shock? Ussing chamber experiments were repeated in physiological buffer devoid of Ca^2+^ or in presence of 50 µM BAPTA-AM to chelate cytosolic Ca^2+^. While the absence of extracellular Ca^2+^ had no impact on the basal and hypotonic current, 50 µM BAPTA decreased the basal and hypotonic current, and almost abolished ENaC hypo shock current (Figure 8). Altogether, the Ca^2+^ imaging and *I*_*sc*_ recording in Ca^2+^-free buffer suggest that the Ca^2+^ rise during hypotonic shock is mainly due to its mobilization from intracellular stores.

As with [Ca^2+^]_i_, transepithelial current increased immediately when the cells were subjected to 20% hypotonic shock. However, maximum transepithelial current was reached much more rapidly (~113 s) than the time needed to reach maximum [Ca^2+^]_i_. In fact, transepithelial current was already decreasing when [Ca^2+^]_i_ reached its maximum, indicating that a low increase in [Ca^2+^]_i_ is sufficient to modulate the current rise. At higher concentrations, Ca^2+^ could decrease ENaC activity. This hypothesis could be in agreement with several reports showing that [Ca^2+^]_i_ in μM concentrations inhibits ENaC open probability [70,71]. At the sustained peak of [Ca^2+^]_i_ after 20% hypotonic shock, Ca^2+^ inhibition of ENaC could explain the transient nature of the current rise detected in alveolar epithelial cells.

In A6 cells, calmodulin has been shown to translocate ENaC at the apical membrane and to increase Na^+^/K^+^-ATPase activity in response to hypotonic shock [27]. We wanted to evaluate if calmodulin, an important transducer of Ca^2+^ function, could be involved in the current rise detected after hypotonic shock. W7, a calmodulin antagonist, did not change basal transepithelial current, but inhibited the current rise after 20% hypotonic shock. These results demonstrate that at basal [Ca^2+^]_i_, calmodulin does not modulate transepithelial current in alveolar epithelial cells. However, calmodulin activates key elements in the current rise when Ca^2+^ is mobilized by exposure to hypotonic shock. Calmodulin is a protein that is very abundant in cells (0.1% of total protein) and has several Ca^2+^-binding domains with different affinities for calcium [72]. It acts as a Ca^2+^ sensor for cell physiology regulation after 0.5 to 5 µM calcium signal transduction [72]. Calmodulin binding to target proteins allows the Ca^2+^ modulation of proteins that cannot bind Ca^2+^ by themselves. The immediate transepithelial current response to hypotonic shock indicates that calmodulin might act on a component directly involved in the rise of transepithelial current. The nature of this component is not known so far. What is the link between Ca^2+^/calmodulin and the role of ENaC and Cl^-^ current in the acute transepithelial current rise reported in the present work? Our data are very similar to the one reported in cerebral astrocyte where calmodulin has been shown to activate hypotonic chloride conductance [73]. In addition to directly stimulate Cl^-^ channel or exchanger, the hypotonic Ca^2+^ mobilization could increase the rapid delivery and membrane insertion of ENaC or Cl^-^ channels. In A549 alveolar epithelial cells, we reported that hypotonic shock promotes a transient increase in the rate of vesicular exocytosis [31]. Since this process is linked to Ca^2+^ elevation [29,74], a reasonable explanation is that hypotonic shock promotes the exocytotic insertion of new channels to the membrane via a Ca^2+^/calmodulin pathway. Several studies report that intracellular Ca^2+^ elevation is involved in apical [75] or basolateral Ca^2+^-dependent exocytosis [76]. It is well known that membrane trafficking plays a major role in modulating ENaC insertion or retrieval [77] and that hypotonic shock favours recycling of ENaC to the apical membrane In A6 cells [78]. Altogether these reports and the data presented here suggest that apical and basolateral Ca^2+^/calmodulin dependent exocytosis could play an important role in modulating the gradual transepithelial current rise induced by hypotonic shock.

20% hypotonic shock induced significant ATP release on the apical side of the monolayers 1 min after stimulation, while no ATP was detected on the basolateral side (Figure 10A). The kinetics and concentrations of ATP release were similar to what has been reported in other experimental protocols [38,79–81]. Two sets of experiments tested if ATP could be implicated in rapid modulation of transepithelial current. First, because ATP has been shown to promote an increase in [Ca^2+^]_i_ and to impact ENaC, we investigated if exogenous ATP, in the absence of osmotic stress, could increase this current. We found that apical, but not basolateral ATP, reduced total transepithelial current by 25%. These results are similar to what was reported in airway epithelial cells [82], trachea [9,83] and alveolar epithelial cells [10,11,84,85]. Because ATP could differentially modulate ionic current during mechanical stress induced by hypotonic shock, *I*_*sc*_ was also recorded after hypotonic shock in the presence of apyrase, an ATP scavenger. Apyrase had no impact on the current rise elicited by hypotonic shock. Altogether, the data presented here ruled out a role for ATP in the rapid transepithelial current increase after hypotonic shock.

In conclusion, our results show that transepithelial Na^+^ current modulation is the result of a complex interplay between K^+^ and Cl^-^ channels, the Na^+^/K^+^-ATPase and KCC in alveolar epithelial cells (Figure 11). KCC is important in Na^+^ transport by recycling K^+^ and allowing apical/basolateral transcellular Cl^-^ transport. During hypotonic shock, the rapid transepithelial current rise is dependent on basolateral Cl^-^ as well as Ca^2+^/calmodulin signaling. In the context of lung pathology, a better knowledge of the channels and transporters that are expressed in alveolar epithelial cells is important for the finding of new therapeutic targets to increase Na^+^ transport in cardiogenic or non-cardiogenic lung oedema. A comprehension of the interactions of Na^+^, Cl^-^ and K^+^ current and transport involved in the cell volume regulation of these cells is also important to understand the repair process following epithelial injury and for the control of cell volume changes during cell growth and apoptosis.
